# Dipicolinic Acid-Tb^3+^/Eu^3+^ Lanthanide Fluorescence Sensor Array for Rapid and Visual Discrimination of Botanical Origin of Honey

**DOI:** 10.3390/foods11213388

**Published:** 2022-10-27

**Authors:** Xijuan Tu, Yunmin Tao, Jiaxu Chen, Chunping Du, Qian Jin, Yuchang He, Ji Yang, Shaokang Huang, Wenbin Chen

**Affiliations:** 1 College of Bee Science, Fujian Agriculture and Forestry University, Fuzhou 350002, China; 2 College of Animal Science, Fujian Agriculture and Forestry University, Fuzhou 350002, China; 3 MOE Engineering Research Center of Bee Products Processing and Application, Fujian Agriculture and Forestry University, Fuzhou 350002, China; 4 College of Food Science, Fujian Agriculture and Forestry University, Fuzhou 350002, China

**Keywords:** honey, botanical origin, lanthanide complex, fluorescence, sensor array

## Abstract

In the present study, a lanthanide fluorescence sensor array was developed for the discrimination of honey’s botanical origin. Dipicolinic acid (DPA) was used as the antenna ligand for sensitizing the fluorescence of Tb^3+^ and Eu^3+^ to prepare the DPA-Tb^3+^/Eu^3+^ complex. This lanthanide fluorescence sensor showed a cross-reactive response to the major constituents of honey, which led to the result that different classes of honey solution exhibited distinct quenching effects on the fluorescence of the DPA-Tb^3+^/Eu^3+^ complex. Furthermore, a fluorescence sensor array composed of ten sensors was constructed by adjusting the pH and the component of the DPA-Tb^3+^/Eu^3+^ complex to show multivariate responses towards honey. The visual fluorescence image of the sensor array was recorded by using a smartphone under excitation with portable UV lamp. Results indicated that the pattern of the visual image was related with the botanical origin. After extracting the RGB value of each sensor in 96-well plate, the ratio of R/G was used for principal component analysis (PCA). The results showed that three classes of honey (astragalus, logan, and litchi) were well distinguished. Moreover, the value of principal component 1 (PC1) showed good linearity with the composition of mixing honey and could be used for semi-quantitative analysis. The proposed lanthanide fluorescence sensor array presents a visual and portable method for the discrimination of a honey’s origin without the use of analytical instruments, and might provide a novel and simple strategy for the measurement of food origin.

## 1. Introduction

Honey, a sweet substance produced by honey bees, is well-recognized as a natural food for nutrition and health [1,2]. The price and functional properties of honey correlate with the origin of the nectar or plant source. Due to the complex of components in honey, the discrimination of a honey’s origin is a great challenge in food authenticity analysis [3,4].

Various analytical methods have been developed for the determination of honey origin. Melissopalynology is the most conventional one, which is based on pollen analysis under optical or electron microscopic [5]. However, sample pretreatment in this technique is time-consuming and the identification requires experience and skill. The analysis of volatile aroma compounds from honey is a more objective method, which is generally performed with GC-MS or electronic nose [6,7,8]. Another strategy is based on the analysis of non-volatile compounds, such as proteins [9], organic acids [10], and phenolics [11], etc., which mostly depends on LC-based techniques. In addition, inorganic microelements are also applied as indicators of honey origin using an inductively coupled plasma-mass spectrometry (ICP-MS) system [12]. Recently, comprehensive non-target information obtained by NMR [13], FT-IR [14,15], fluorescence [16,17], or high-resolution mass spectrometry (HRMS) [18] has also been developed to differentiate honey origin. Despite this progress, sophisticated and expensive analytical instruments and time-consuming sample pretreatment are generally required in the reported methods. In the present work, a simple, green, and instrument-free analytical method based on a lanthanide fluorescence sensor array was developed for rapid and visual discrimination of honey origin.

Array-based sensing system, which generally uses a number of cross-reactive sensors to generate a distinct pattern response, has gained intensive interest in sensing complex analytes [19]. By using pattern recognition algorithms, the cross-reactive array response could provide distinguished information for analytes without sophisticated design and the synthesis of specific sensors. In recent years, array-based sensors, particularly fluorescent array sensors, have been recognized as a fast, sensitive, portable, and inexpensive sensing technique and applied in a broad range of applications including environmental analysis [20,21], biomedical diagnosis [22,23], and food control [24]. In the present study, we provide a proof-of-concept that simply constructed dipicolinic acid (DPA)-Tb^3+^/Eu^3+^ lanthanide sensor array can be used for rapid, visual, and instrument-free discrimination of honey origin.

## 2. Materials and Methods

### 2.1. Materials

Terbium chloride hexahydrate (TbCl_3_·6H_2_O), europium chloride hexahydrate (EuCl_3_·6H_2_O), glucose, fructose, vitamin B_6_, vitamin B_2_, vitamin C, kaempferol, quercetin, caffeic acid, p-coumaric acid, and vanillic acid were obtained from Aladdin (Shanghai, China). Dipicolinic acid (DPA), proline, gluconic acid, citric acid, succinic acid, MgCl_2_, ZnCl_2_, CuCl_2_, and MnCl_2_ were obtained from Macklin (Shanghai, China). Other reagents were all of analytical grade. Honey samples were directly collected from apiaries during the period of flowering. Litchi (*Litchi chinensis*) and longan (*Dimocarpus longan*) honey samples were collected in Fujian province, and astragalus (*Astragalus membranaceus*) honey samples were collected in Gansu province. Ultrapure water (18.2 MΩ·cm) was used in all the experiments.

### 2.2. Preparation of DPA-Tb^3+^/Eu^3+^ Complex Sensors

Stock solutions of TbCl_3_ (20 µM), EuCl_3_ (50 µM), DPA (500 µM), and borate buffer (pH 7.4 and pH 8.4, 200 mM) were prepared in water. Then, these aqueous solutions were mixed and diluted by water to the final concentration as described in Table S1 for the preparation of lanthanide complex sensors.

### 2.3. Effects of Honey on the Fluorescence of DPA-Tb^3+^/Eu^3+^ Complex

Three milliliters of DPA-Tb^3+^/Eu^3+^ complex solution was transferred into a cuvette, then 10 µL of honey aqueous solution (0.1 g/mL) was added at a time until a total titration volume of 80 µL. Fluorescence spectra were recorded by Shimadzu RF5301 with an excitation wavelength of 280 nm, slit of excitation and emission were 3 nm and 5 nm, respectively, the scan range was from 300 nm to 650 nm. Quenching efficiency (QE) was calculated by QE = (F_0_ − F)/F_0_, where F_0_ and F were the fluorescence intensity without and with the presence of honey solution, respectively.

### 2.4. Cross-Reactive Response of DPA-Tb^3+^/Eu^3+^ Complex to Honey Constituents

Stock solutions of kaempferol, quercetin, p-coumaric acid, vanillic acid, and caffeic acid were prepared in methanol. In addition, solutions of glucose, fructose, vitamin B_6_, vitamin B_2_, vitamin C, proline, gluconic acid, citric acid, succinic acid, MgCl_2_, ZnCl_2_, CuCl_2_, and MnCl_2_ were prepared in water. After 3 mL of DPA-Tb^3+^/Eu^3+^ complex solution was transferred into a cuvette, fluorescence titration and spectra recording were performed as described in Section 2.3. The simulated concentrations are listed in Table S2.

### 2.5. Sensor Array Measured by Microplate Reader

Three hundred microliters of sensor solution were transferred into 96-well plate, then 80 µL of honey solution were added into each well. The fluorescence spectra of the array were recorded using Thermo Varioskan LUX microplate readers with excitation wavelength of 280 nm. The emission intensity at 488 nm, 544 nm, and 614 nm was used for the principal component analysis (PCA) performance.

### 2.6. Visual Discrimination Using Sensor Array

The sensor array was prepared as described in Section 2.5. After the addition of 80 µL of honey solutions, the array was put into portable UV lamp box under lights of 254 nm and 365 nm. Fluorescence image was recorded by a smartphone (Huawei JKM-AL00b) positioned on the top of the lamp box, the values of RGB in each well were extracted using Photoshop software, and the obtained values were applied for the PCA performance.

## 3. Results and Discussion

**Figure 1:**
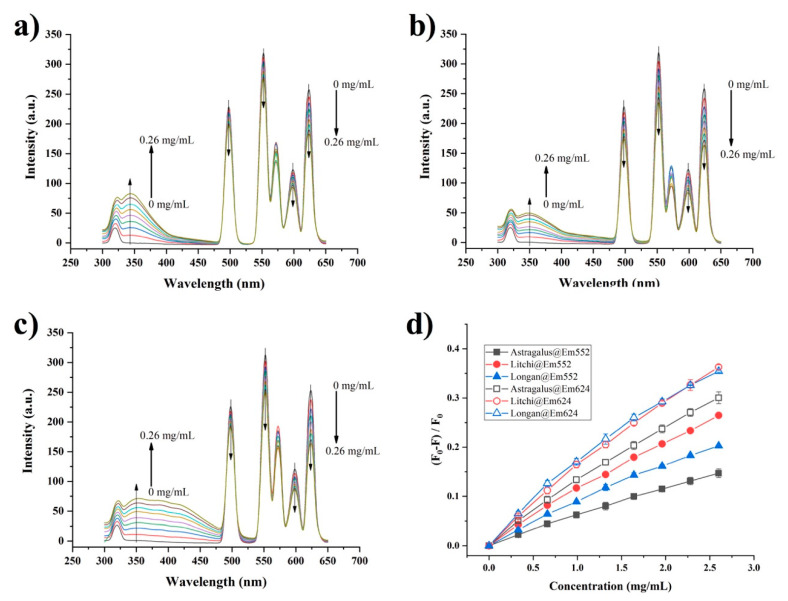
Fluorescence spectra of DPA-Tb^3+^/Eu^3+^ complex with the presence of aqueous honey solution (**a**) astragalus honey, (**b**) litchi honey, (**c**) longan honey. Comparison of quenching efficiency at Em552 and Em624 by three honey solutions (**d**). F_0_ and F represents the fluorescence intensity without and with the presence of honey solution, respectively. Error bar presents the standard deviation of triplicate experiments.

Quenching on the fluorescence of the DPA-Tb^3+^/Eu^3+^ complex was observed when the aqueous honey solution was introduced. Dipicolinic acid was selected as the antenna ligand for the preparation of the lanthanide complex because of its sensitizing ability for both Tb^3+^ and Eu^3+^ [25,26]. As shown in Figure 1, the fluorescence spectra of the DPA-Tb^3+^/Eu^3+^ complex exhibit typical luminescence emissions from both Tb^3+^ and Eu^3+^. The emissions centered at 497 nm (Em497) and 552 nm (Em552) are attributed to the transitions of Tb^3+^ from ^5^D_4_ to ^7^F_6_ and ^7^F_5_, respectively [27], while emissions at 598 nm (Em598) and 624 nm (Em624) are from the ^5^D_0_ to ^7^F_1_ and ^7^F_2_ transitions of Eu^3+^, respectively [27]. Interestingly, when the aqueous solution of honey was added to the DPA-Tb^3+^/Eu^3+^ complex, the fluorescence emissions from lanthanide ions were significantly quenched. As observed in Figure 1a, when astragalus honey solution was added, the fluorescence intensity of both Tb^3+^ and Eu^3+^ were dramatically decreased. This fluorescence quenching was also observed in the titration with the other two classes of honey, longan and litchi (Figure 1b,c). The comparison of quenching efficiency (QE) on the emission intensity of Em552 and Em624 is shown in Figure 1d. It can be clearly observed that the QE on Em624 induced by the three classes of honey was higher than that on Em552. For example, as the concentration of honey was increased to 2.6 mg/mL the QE on Em552 ranged from 0.15 to 0.26, while on Em624, the QE ranged between 0.30 and 0.36. Moreover, the QE induced by litchi honey was higher than that of longan honey, while that induced by astragalus honey showed the lowest level. These results indicated that quenching performance on DPA-Tb^3+^/Eu^3+^ complex was related to the botanical origin of honey.

Additionally, an appearance of fluorescence peak located near 350 nm was observed with the addition of the honey solution (Figure 1a–c). This can be ascribed to the intrinsic fluorescence of honey, which may result from protein and phenolic constituents [28]. It was valuable to notice that each class of honey exhibited its characterized spectra of intrinsic fluorescence. Astragalus honey showed an intense fluorescence peak centered at 343 nm. For litchi and longan honey, the emission peaks moved to 350 nm. Furthermore, a small shoulder peak near 416 nm was found in both litchi honey and longan honey, and the intensity in longan honey was much stronger than that in litchi honey. These above results illustrate the distinct emission profiles when the DPA-Tb^3+^/Eu^3+^ complex interacted with different honeys, which could provide multiple fluorescence information for distinguishing the botanical origins of honey.

**Figure 2:**
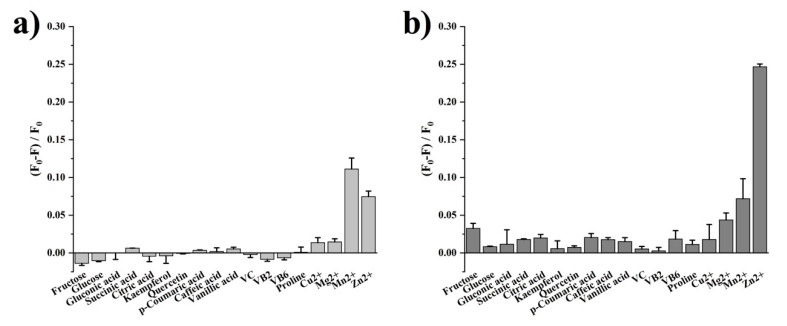
Comparison of effect on (**a**) Em552 and (**b**) Em624 from eighteen major constituents in honey. The simulated concentration of each constituent is listed in Table S2. F_0_ and F represent the fluorescence intensity without and with the presence of constituent, respectively. Error bar presents the standard deviation of triplicate experiments.

The DPA-Tb^3+^/Eu^3+^ complex exhibits a cross-reactive response to the major constituents of honey. Eighteen constituents generally found in honey, including sugars, organic acids, vitamins, phenolics, and mineral cations, were studied with simulated concentration as listed in Table S2 according to the reported literature [1,29,30,31,32,33]. The results of fluorescence titration are summarized in Figure 2. Sugar is the main component of honey, in which the fructose content is between 30% and 45% (*w*/*w*), and glucose is in the range from 24% to 40% (*w*/*w*) [1]. We prepared fructose and glucose solution with a concentration of 40% and 30% (*w*/*w*), respectively, to simulate the sugar composition in honey and studied their effects on the fluorescence of the DPA-Tb^3+^/Eu^3+^ complex. Results shown in Figure 2a indicate that both the fructose and glucose solution show a slight enhancing effect on Em552, whereas, under the same assay, the quenching of Em624 by fructose and glucose was observed. Moreover, the intensity of Em624 was decreased by about 3.2% by fructose, which was larger than the QE by glucose.

Organic acids make an important contribution to the acidity and antibacterial activities of honey [10]. The profile of organic acids in honey has been reported to be related to the botanical origin of honey [10]. Generally found organic acid compounds in honey include gluconic acid, succinic acid, and citric acid, etc. [30]. Gluconic acid is the major organic acid in honey, which is mainly produced as a result of the oxidation of glucose by glucose oxidase. It can be seen from Figure 2a that gluconic acid has no obvious effect on the fluorescence of Tb^3+^. Compared with gluconic acid, succinic acid showed slight quenching on Em552, while citric acid exhibited a slight enhancing effect. For Em624, the results (Figure 2b) indicated that all three organic acids showed a quenching effect, and the QEs of succinic acid and citric acid were similar and both larger than gluconic acid.

Phenolic compounds are classical markers for the discrimination of honey origin [11]. The effects of five typical phenolic components in honey, p-coumaric acid, caffeic acid, vanillic acid, kaempferol, and quercetin, on the fluorescence of the DPA-Tb^3+^/Eu^3+^ complex were investigated. The results showed that the fluorescent complex displayed slight responses to different phenolic compounds. For instance, kaempferol slightly enhanced the fluorescence of Tb^3+^, but the other four phenolic compounds quenched the intensity, with QE of less than 1%. Besides, all five phenolic compounds showed a quenching effect on Em624, in which p-coumaric acid showed the highest QE of 2%.

Honey is rich in vitamins, particularly in vitamin C, vitamin B_2_ and vitamin B_6_ content, with between 2.2~2.5 mg/100 g, 0.01~0.02 mg/100 g, and 0.01~0.32 mg/100 g, respectively [1]. These vitamins showed enhancement on Em552 while quenching on Em624. In addition, the QE induced by vitamin B_6_ was higher than that of vitamin C and vitamin B_2_.

Proline is used as an indicator for honey ripeness, and its content should be more than 180 mg/kg [34]. The fluorescence of Em552 was almost unchanged after the introduction of proline. In contrast to Em552, the fluorescence of Em624 was slightly quenched about 1% by proline.

In addition to the organic components, mineral ions including Mg^2+^, Zn^2+^, Cu^2+^, and Mn^2+^ were also investigated. As shown in Figure 2, all these four ions show significant quenching on both Em552 and Em624. Of note, Mn^2+^ and Zn^2+^ achieved the highest QE on the fluorescence of Tb^3+^ and Eu^3+^, respectively. Furthermore, Cu^2+^ and Mg^2+^ showed similar QE on Em552, but for Em624, Mg^2+^ exhibited higher QE than Cu^2+^.

The above results indicate that the main constituents in honey elicit different signal responses from the DPA-Tb^3+^/Eu^3+^ complex. The fourteen organic compounds caused slight variance of fluorescence of Tb^3+^, while the four inorganic cations led to significant fluorescence quenching. Furthermore, the fluorescence of Eu^3+^ was more sensitive to the constituents of honey. All the eighteen compounds resulted in quenching of Em624, and the cations contributed to the major quenching performance. In addition, fructose, organic acids, phenolics, and vitamin B_6_ also induced significant quenching of emissions from Eu^3+^. Therefore, the lanthanide fluorescence sensor DPA-Tb^3+^/Eu^3+^ complex performed multiple and cross-reactive signal responses to the honey constituents. This means that the botanical information of honey may be presented through the interaction between the lanthanide sensor and honey constituents. This could provide a novel and simple method for the identification of honey’s botanical origin.

**Figure 3:**
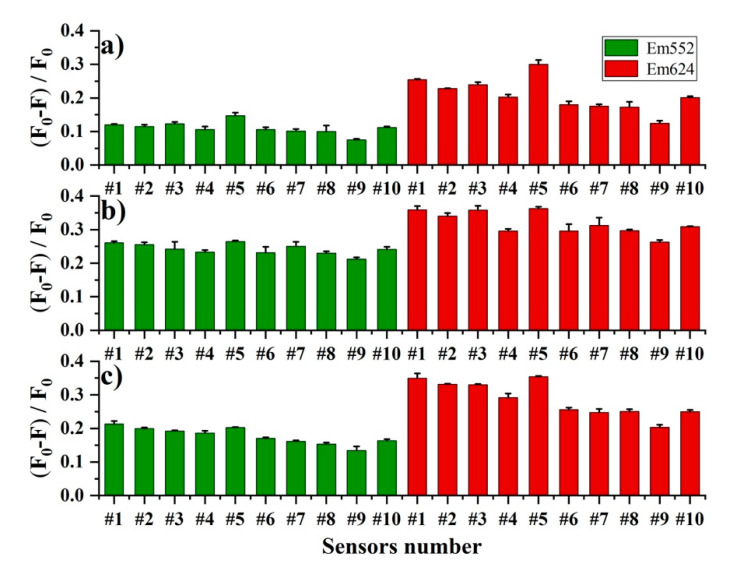
Fluorescence response of the ten sensors (#1–10) in the constructed sensor array induced by three classes of honey: (**a**) astragalus honey; (**b**) litchi honey; (**c**) longan honey. The final concentration of honey was 2.60 mg/mL. F_0_ and F represent the fluorescence intensity without and with the presence of honey solution, respectively. Error bar presents the standard deviation of triplicate experiments.

To obtain a multivariate fluorescence response towards honey, a fluorescence sensor array was constructed by simply adjusting the ratio of lanthanide ions to the ligand and the pH of the solution. The detailed conditions of the sensor array are shown in Table S1, and the specific fluorescence responses at Em552 and Em624 of the ten sensors are summarized in Figure 3. The results indicated that the QE of the sensors was in different ranges when reacting with the three classes of honey. For instance, the QE on Em552 caused by astragalus honey was between 0.08 and 0.15, while for litchi and longan honey, the QE was improved to 0.21~0.26 and 0.13~0.21, respectively. The distribution range was in the order of longan > astragulas > litchi. The QE on Em624 was in a wider range compared with that on Em552. For example, the QE caused by astragalus was distributed from 0.12 to 0.30, and the order of the distribution range was changed to astragulas > longan > litchi. Meanwhile, it was clearly found that the QE at pH 7.4 (sensor #1–5) was higher than at pH 8.4 (sensor #6–10). Particularly, sensor #9 showed the lowest QE of all three classes of honey, while sensor #5 showed the highest QE by astragulas and litchi, except for longan honey, where the highest QE at Em552 was achieved in sensor #1. Furthermore, it could be seen from sensors #1–3 that, with the decrease in Tb^3+^ concentration in the DPA-Tb^3+^/Eu^3+^ complex, the QE at Em552 was reduced when reacted with litchi and longan honey, while, as with astragalus honey, QE was slightly varied in sensors #1–3. For Em624, the QE was also decreased with the reduction of Tb^3+^ concentration in the DPA-Tb^3+^/Eu^3+^ complex. Comparing sensors #4, #2, and #5, in which the concentrations of DPA were decreased in the complex, the QE at Em552 and Em624 were both increased. Moreover, the increment of QE was in the order of astragulus > litchi > longan. These results indicate that the three classes of honey exhibited different QEs on the ten sensors, which made the array generate unique multivariate responses related to botanical origin.

**Figure 4:**
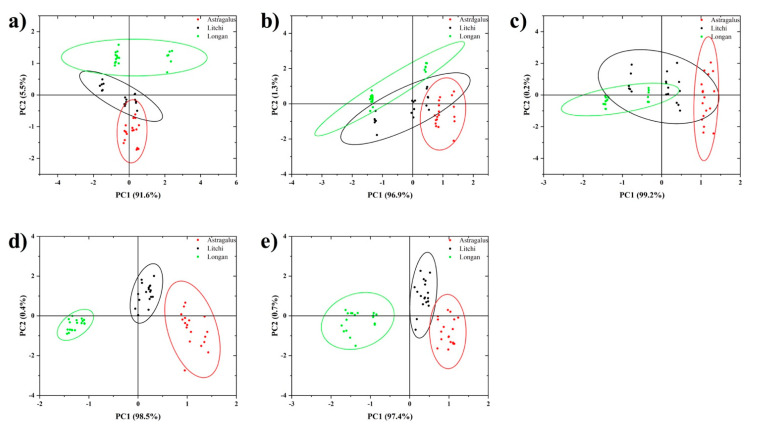
PCA results of sensor array measured using microplate reader. Data obtained by (**a**) Em488, (**b**) Em544, (**c**) Em614, (**d**) Em614/Em488, and (**e**) Em614/Em544.

The fluorescence of the array was quickly measured using microplate reader to check if this constructed multivariate response could be used for the discrimination of honey origin. The emissions at 488 nm and 544 nm from Tb^3+^ and the emissions at 614 nm from Eu^3+^ recorded by microplate reader were used to establish PCA discrimination. When emission intensity was used for PCA analysis, longan honey and astragalus honey could be separated (Figure 4a–c). However, an overlap was observed between litchi honey and the other two classes. To improve the PCA separation, intensity ratios of emission from Eu^3+^ to emission from Tb^3+^ (Em614/Em488 and Em614/Em544) were used instead to reduce the background differences between plate wells. The results indicated that the separation was improved significantly after ratio transform (Figure 4d,e). Therefore, as proved above, this multivariate signal response generated by the cross-reactive DPA-Tb^3+^/Eu^3+^ fluorescence sensor array could be applied for the discrimination of honey origin.

**Figure 5:**
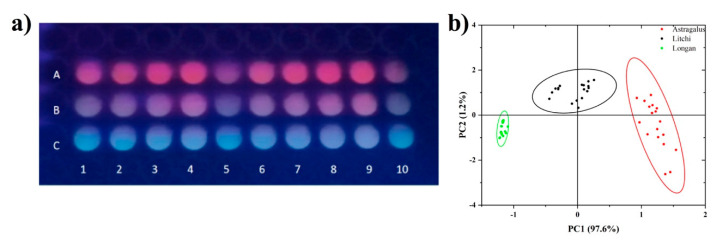
(**a**) Fluorescence image of sensor array (1–10: sensor number) with the presence of honey (A: astragalus honey; B: litchi honey; C: longan honey). (**b**) PCA results obtained by the ratio of color value R/G.

**Figure 6:**
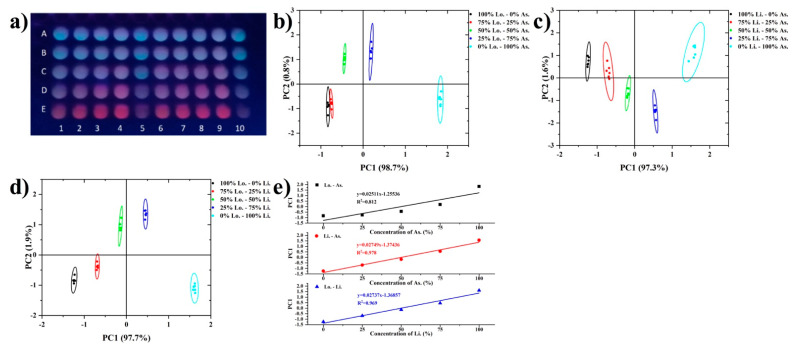
(**a**) Fluorescence image of sensor array (1–10: sensor number) response to longan–astragalus mixing honey (A: 100–0%; B: 25–75%; C: 50–50%; D: 75–25%; E: 0–100%). PCA results of honey mixture: (**b**) longan–astragalus mixing (Lo.-As.); (**c**) litchi–astragalus mixing (Li.-As.); (**d**) longan–litchi mixing (Lo.-Li.). (**e**) linear fit of PC1 with mixing concentration in honey mixture.

We further demonstrated that this constructed lanthanide fluorescence sensor array provided visual image patterns for the discrimination of honey origin. Since the fluorescence emissions from Tb^3+^ and Eu^3+^ were in the visual colors of green and red, respectively, it was reasonable to speculate that the multivariate response of the sensor array towards honeys would also make the array present unique visual images. Figure 5a shows the fluorescence image of the sensor array under a portable UV lamp with the presence of honeys. It is clearly seen that honey from different botanical origins showed characteristic visual array images. After taking photos by smartphone, the RGB color value of each plate well was read by software, then PCA was performed with the ratio of R/G. The PCA results illustrated that the three classes were well distinguished (Figure 5b). This visual method was further applied to discriminate the mixture of different honeys. As shown in Figure 6a, the mixtures of astragalus honey and longan honey with different concentrations displayed distinct array images. Furthermore, it can be seen from Figure 6b–d that the mixtures of each two classes of honey are all distinguished. Principal component 1 (PC1), it was observed, accounts for most of the components in all the mixing conditions. Thus we used the average score value of PC1 to fit with the mixing concentration, and it was interesting to find that good linearity was obtained in each mixing experiment (Figure 6e). This means that semi-quantitative analysis of honey mixing could be achieved based on the score of PC1. Compared with instrument analysis methods, this rapid visual fluorescence sensor array method is very simple, green, and low-cost. Moreover, the fluorescence image can be recorded by using a smartphone. Considering that apiary generally far away from urban areas, such a simple discrimination method would be suitable for fast in-field analysis.

## 4. Conclusions

In summary, we have developed a lanthanide fluorescence sensor array for rapid visual discrimination of honey origin. Fluorescence quenching of the DPA-Tb^3+^/Eu^3+^ complex was observed when reacted with aqueous honey solution. This quenching effect was demonstrated to result from the cross-reactive response of the DPA-Tb^3+^/Eu^3+^ complex to multiple constituents of honey. Then, a fluorescence sensor array was constructed by changing the pH and the component of the DPA-Tb^3+^/Eu^3+^ complex. The fluorescence response of the sensor array towards honey was related with the botanical origin of honey, and the fluorescence image of the sensor array provided a visual response pattern for the discrimination of honey origin. The proposed visual array method is simple, rapid, and without the use of analysis instruments, and could provide a new strategy for the portable and fast in-field measurement of honey origin. In future research, the dimensions and diversity of the sensor array could be enlarged to distinguish more classes of honey and to classify geographic origin.

## Data Availability

Data are contained within the article.

## Supplementary Materials

The following supporting information can be downloaded at: https://www.mdpi.com/article/10.3390/foods11213388/s1, Table S1: Final concentration of ten DPA-Tb^3+^/Eu^3+^ complex sensors; Table S2: Simulated content of eighteen major constituents in honey.
