# Supplementary material for: Dipicolinic Acid-Tb3+/Eu3+ Lanthanide Fluorescence Sensor Array for Rapid and Visual Discrimination of Botanical Origin of Honey
Source: Foods. 2022 Oct 27;11(21):3388. doi: 10.3390/foods11213388 (PMC9656745; doi:10.3390/foods11213388)
Supplement: Supplementary file 1 [file foods-11-03388-s001.zip › foods-1981566-supplementary.pdf]

foods MDPI

Supplementary Materials

Dipicolinic acid-Tb^{3+}/Eu^{3+} lanthanide fluorescence sensor array for rapid and visual discrimination of botanical origin of honey

Xijuan Tu [1,3,4,†], Yunmin Tao [1,2,†], Jiaxu Chen [1,3], Chunping Du [1,3], Qian Jin [1,3], Yuchang He [1,3], Ji Yang [1,3], Shaokang Huang [1,3], Wenbin Chen [1,3,4,*]

1 College of Bee Science, Fujian Agriculture and Forestry University, Fuzhou 350002, China.
2 College of Food Science, Fujian Agriculture and Forestry University, Fuzhou 350002, China.
3 College of Animal Science, Fujian Agriculture and Forestry University, Fuzhou 350002, China.
4 MOE Engineering Research Center of Bee Products Processing and Application, Fujian Agriculture and Forestry University, Fuzhou 350002, China.
* Correspondence: wbchen@fafu.edu.cn
† These authors contributed equally to this work.

Table S1. Final concentration of ten DPA-Tb^{3+}/Eu^{3+} complex sensors

Sensor number	$EuCl_3$ (μM)	$TbCl_3$ (μM)	DPA (μM)	Borate buffer (pH / mM)
Sensor#1	3.3	1.2	13	pH 7.4 / 50
Sensor#2	3.3	0.8	13	pH 7.4 / 50
Sensor#3	3.3	0.5	13	pH 7.4 / 50
Sensor#4	3.3	0.8	16	pH 7.4 / 50
Sensor#5	3.3	0.8	10	pH 7.4 / 50
Sensor#6	3.3	1.2	13	pH 8.4 / 50
Sensor#7	3.3	0.8	13	pH 8.4 / 50
Sensor#8	3.3	0.5	13	pH 8.4 / 50
Sensor#9	3.3	0.8	16	pH 8.4 / 50
Sensor#10	3.3	0.8	10	pH 8.4 / 50

Table S2. Simulated content of eighteen major constituents in honey

Constituent	Content in honey	Simulated content
Fructose	30-45% (w/w) [1]	40%
Glucose	24-40% (w/w) [1]	30%
Gluconic acid	649.0-6475.1 mg/kg [29]	1500 mg/kg
Succinic acid	17-187 mg/kg [30]	150 mg/kg
Citric acid	48-351 mg/kg [30]	150 mg/kg
p-Coumaric acid	1.5-11.8 mg/kg [31]	5 mg/kg
Caffeic acid	0.03-2.29 [32]	5 mg/kg
Vanillic acid	0.89-5.91 mg/kg [31]	5 mg/kg
Kaempferol	0.45-3.92 mg/kg [31]	5 mg/kg
Quercetin	0.61-2.43 mg/kg [31]	5 mg/kg
Vitamin C	2.2-2.5 mg/100g [1]	2.5 mg/100g
Vitamin B_2	0.01-0.02 mg/100g [1]	0.02 mg/100g
Vitamin B_6	0.01-0.32 mg/100g [1]	0.02 mg/100g
Proline	229-665 mg/kg [33]	400 mg/kg
Mg	0.7-13 mg/100g [1]	10 mg/100g
Zn	0.05-2 mg/100g [1]	1 mg/100g
Cu	0.02-0.6 mg/100g [1]	0.2 mg/100g
Mn	0.02-2 mg/100g [1]	1 mg/100g
